# The Effect of 5′-Adenylic Acid on Hepatic Proteome of Mice Radiated by ^60^Co γ-ray

**DOI:** 10.3390/ijms15010186

**Published:** 2013-12-24

**Authors:** Cuilin Cheng, Haitian Zhao, Zhenyu Wang, Weihong Lu, Lu Wang, Rongchun Wang, Lei Yao

**Affiliations:** 1 School of Food Science and Engineering, Harbin Institute of Technology, Harbin 150090, China; E-Mails: ccuilin@hit.edu.cn (C.C.); zhaoht9999@gmail.com (H.Z.); lwh@hit.edu.cn (W.L.); hitwanglu@hit.edu.cn (L.W.); wangrongchun@hit.edu.cn (R.W.); yaoleiyl2000@gmail.com (L.Y.); 2 Institute of Extreme Environmental Nutrition and Protection, Harbin Institute of Technology, Harbin 150090, China; 3 School of Forestry, Northeast Forestry University, Harbin 150026, China; 4 School of Food Science and Engineering, Northeast Agriculture University, Harbin 150030, China

**Keywords:** 5′-AMP, protective effect, radiation damage, proteomics

## Abstract

Understanding the protection mechanism of 5′-AMP requires comprehensive knowledge of the proteins expressed during the period that the body is exposed to irradiation. Proteomics provides the tools for such analyses. Here, the experimental ICR mice were divided into three groups (normal group, model group and 5′-AMP + irradiation group). After different treatment, the hepatic total protein of each animal in three groups was separated by two-dimensional gel electrophoresis (2-DE). 2-DE analysis revealed fifty-eight protein spots were differentially expressed in comparison to three groups. From 58 protein spots, we selected nine spots to identify by MALDI-TOF-MS and received credible results. They were determined to be type I arginase, annexin A5, regucalcin, catalase, Tpm3 protein, Pdia4 protein, 14-3-3 protein epsilon, NAD-Malate dehydrogenase and heat shock protein 90. Considering the characteristic of these proteins, we proposed a possible protection pathway.

## 1. Introduction

Proteins act as gene-encoded products to execute and reflect various biological functions in living organisms. It is more immediate and efficient to analyze the expression of genes at the protein molecule level. By comparing proteome difference between tissues or cells in various pathological and physiological conditions, biomarkers related to diseases can be selected and pathogenesis determined. Thus, comparative analysis of differentially expressed proteins is valuable for the therapy of various diseases including radiation damage.

The body’s cells exposed to irradiation would start a complex network process to decide the fate. The irradiation-related genes are activated to transmit the information between and within cells via various signal pathways, which in turn induces expression level differences in several target proteins, including cell cycle regulation, cell growth, cell apoptosis, DNA damage repair, *etc.* [1–3]. Based on preliminary experiments, we have confirmed that 5′-adenylic acid (5′-AMP) has a protective effect against irradiation. However, it’s not entirely clear which enzymes play key roles in this complex signal-transduction process. Proteomics technologies provide important clues for a better understanding about these genes as well as scientific evidence for the mechanism of 5′-AMP against radiation.

As an important metabolic organ, the liver contains almost all of the enzymes in the body involved in metabolic activities of many endogenous and exogenous compounds. Moreover the liver is often incidentally irradiated during whole abdomen or whole body radiation therapy (RT). Liver damage caused by radiation becomes the main limiting factor to radiotherapy applied in tumor treatment. For the statement above, we proposed the liver as the appropriate study object for finding radiosensitive proteins and detecting the target molecule of radioprotector (5′-AMP). Based on earlier animal experiments, we have found that 5′-AMP could regulate oxidation-reduction state in liver (results not shown), showing the protective effect against irradiation. To find the target molecules of 5′-AMP, a proteomic approach (two-dimensional gel electrophoresis (2-DE) and mass spectrometry (MS)) was used for seeking differential protein expression in radiated-mice liver treated by 5′-AMP. To date, no proteomics studies of liver tissue from 5′-AMP treated mice have been reported yet. The purpose of this study was to identify new protein biomarkers so as to help understand the protection mechanisms of 5′-AMP against radiation. This experimental data would provide an experimental basis for using 5′-AMP as a lead compound to design and synthesize the new antiradiation drugs.

## 2. Results and Discussion

### 2.1. Effect of 5′-AMP on Cell Apoptosis of Liver Tissue Induced by γ-ray Radiation

**Figure 1.**
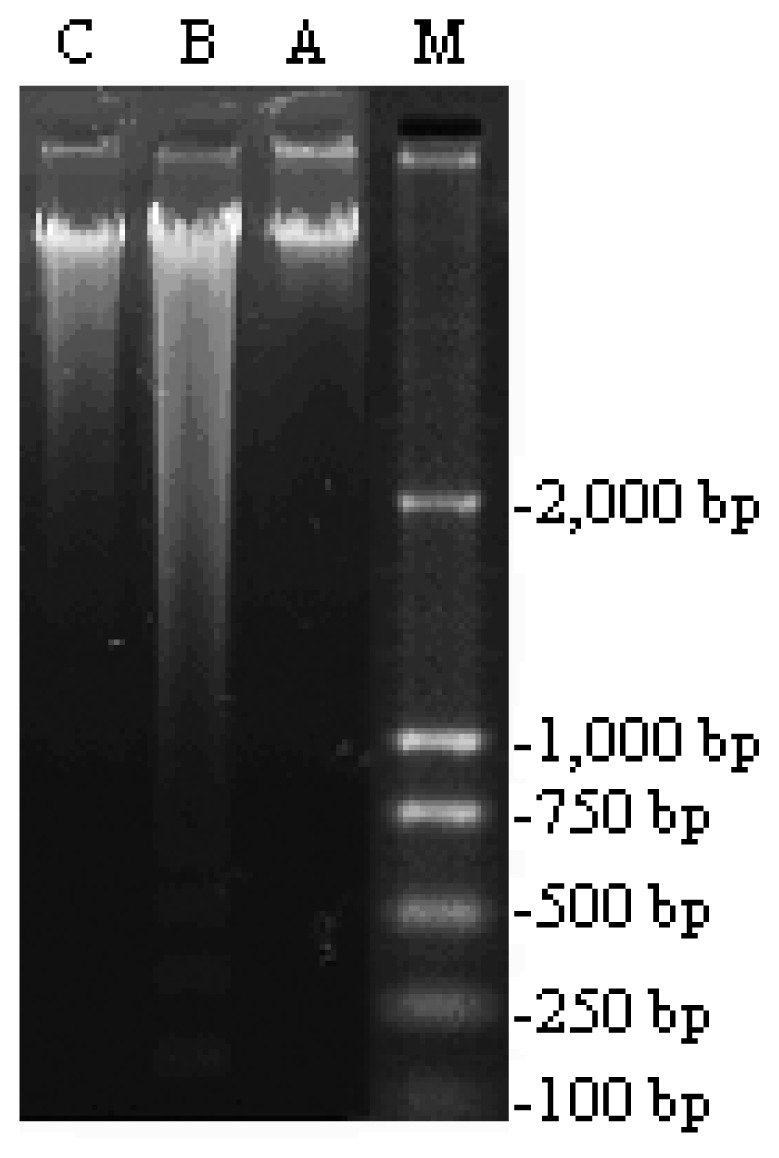
Agarose gel showing cell apoptosis by ^60^Co γ-ray radiation and preventive effect of 5′-AMP (0.16 g/kg·bw/day) on liver tissue of radiated mice. Lanes (from left) 5′-AMP-treated group (**C**), model group (**B**), normal group (**A**), molecular weight marker (**M**).

As shown in Figure 1, irradiation induced DNA damages with typical apoptosis features of “ladder”. The results demonstrated that 4 Gy ^60^Co γ-ray radiation induced apoptosis in normal liver cells. The supplement of 5′-AMP at the dose of 0.16 g/kg·bw/day in diet reversed the DNA damages and reduced the apoptosis levels comparable to controls.

### 2.2. Effect of 5′-AMP on Protein Expression Profile in Radiated Mice

**Figure 2.**
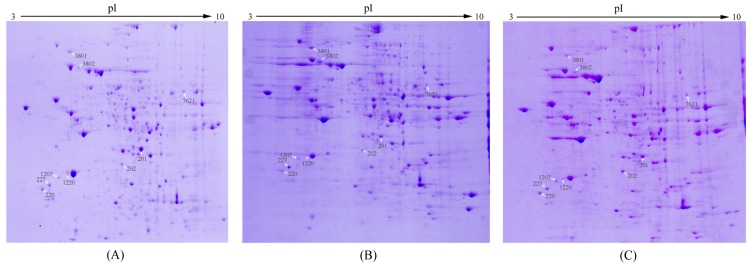
2-DE gel images from normal group (**A**); model group (**B**); and 5′-AMP-treated group (**C**). The gel images were the representative gel of nine replicate gels collected from three independent experiments. The white arrows showed the differentially expressed spots chosen for MALDI-TOF MS/MS analysis.

In the same sample amount and electrophoretic conditions, 2-DE and gel stain were conducted to investigate the differential protein expression of three groups (Normal group: no radiation + no treatment; Model group: radiation alone (4 Gy ^60^Co γ-ray); AMP-treated group: radiation (4 Gy ^60^Co γ-ray) + 5′-AMP (0.16 g/kg·bw/day)). Representative 2-DE gel images for hepatic proteins of three groups are shown in Figure 2. Gel images were analyzed via PDQuest 2-D analysis software. The distribution of three groups of total protein spots was clear and similar. The numbers of protein dots in three groups were 551, 230 and 260 spots in normal group, model group and 5′-AMP treated group respectively. From these numbers, it was observed that the total protein dots of mice radiated by 4 Gy γ-ray decreased, which indicated that irradiation changed protein expression in liver. Moreover, there were 209 spots expressed in all three groups and 157 spots only in normal group.

**Figure 3.**
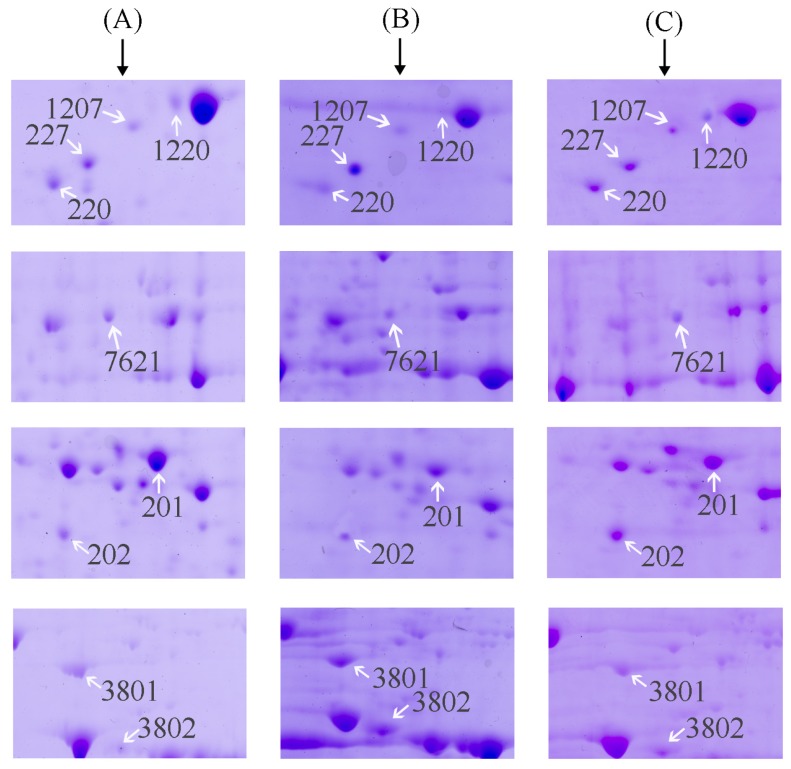
The expanded region of differentially expressed protein spots. The regions were cropped from the representative gel as shown in Figure. 2. The proteins shown by arrow were the differentially expressed proteins. (**A**) Normal group; (**B**) Model group; and (**C**) AMP-treated group.

Gel images detected about 500 protein spots in each gel. Fifty-eight protein spots were found to be significantly regulated in the model group compared with normal (*p* < 0.05). Nine of fifty-eight spots exhibited 1~2-fold increase or decrease in abundance as observed in all replicate gels. These nine protein spots were cut from the gels and further identified by MALDI-TOF MS/MS analysis. The nine regulated proteins were indicated by the arrowed spots in Figure 2 and by the expanded plots in Figure 3.

### 2.3. Identification of the Differentially Expressed Proteins

Expression of the selected protein spots in three groups are listed in Table 1. Among the nine selected proteins, there were six proteins (spot 201, 1207, 1220, 7621, 220 and 202), which showed significantly down-regulated expression (*p* < 0.01) in the model group, compared to the normal group. But treatment with 5′-AMP before radiation increased the expression of the six proteins (*p* < 0.05 or *p* < 0.01), compared to the model group. Another three proteins (spot 227, 3802 and 3801), in contrast, showed significantly increased expression in the model group (*p* < 0.01), compared to the normal group. And treatment with 5′-AMP before radiation decreased the expression of the three proteins (*p* < 0.05 or *p* < 0.01), compared to the model group.

**Figure 4.**
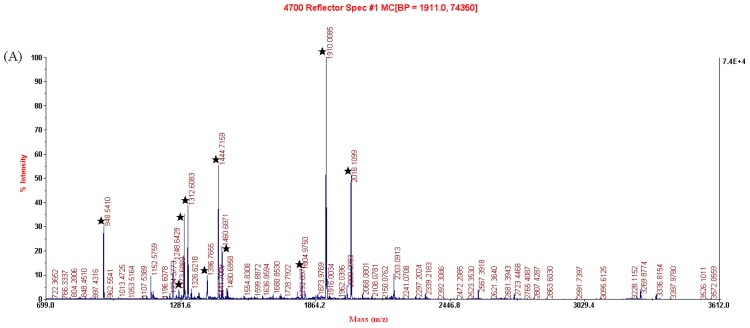

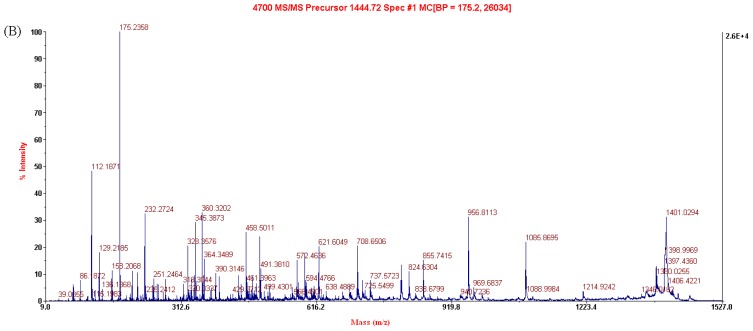
MALDI-TOF MS/MS spectra of the spot 201 cut from the 2-DE gel. (**A**) Peptide mass fingerprint (PMF) of the tryptic digest of spot 201. Peptide signals identified were marked with asterisks; and (**B**) MS/MS profile of the peptide with a mass of 1444.72 Da.

Each selected protein was identified using MALDI-TOF MS/MS. The result of analysis of spot 201 is shown in Figure 4 as an example. The results of the identification of the selected nine protein spots are listed in Table 1. They were identified as (1) spot 201: Type I arginase (AI); (2) spot 1207: Annexin A5; (3) spot 1220: Calmodulin (CaM); (4) spot 7621: Catalase; (5) spot 227: Tpm3 protein; (6) spot 3802: Pdia4 protein; (7) spot 220: tyrosine 3-monooxygenase/tryptophan 5-monooxygenase activation protein, epsilon polypeptide (14-3-3 protein epsilon); (8) spot 202: NAD-Malate dehydrogenase (NAD-MDH); and (9) spot 3801: Heat shock protein 90 (Hsp90).

### 2.4. Validation of Differentially Expressed Protein

To validate the results of 2-DE, we investigated the expression of Hsp90 and catalase, using western blot and biochemical method respectively.

#### 2.4.1. Western Analysis of Hsp90

**Figure 5.**
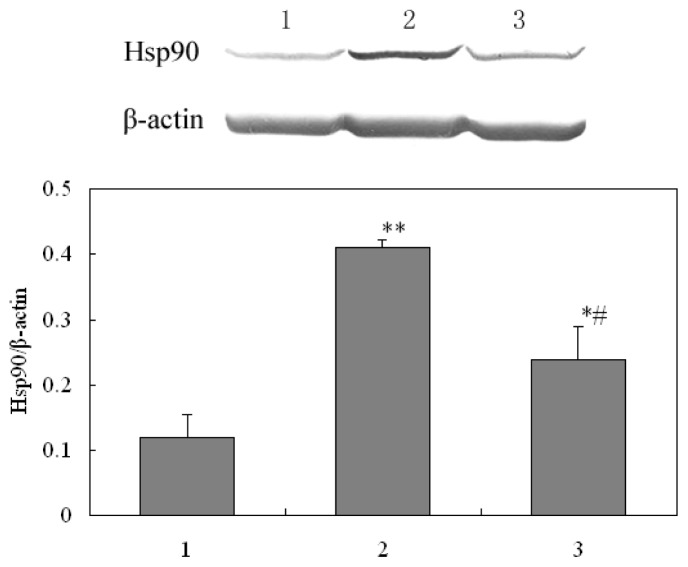
Western blot image of Hsp90 expression and β-actin (loading control) protein expression. The expression of Hsp90 in each sample from three groups is internally normalized to β-actin and the values are presented underneath the image. (**1**) Normal group; (**2**) Model group; and (**3**) 5′-AMP-treated group. ** *p* < 0.01, * *p* < 0.05 *vs.* Normal; ^#^ *p* < 0.05 *vs.* Model.

Hsp90 was selected for validation test. Western blot image and the percent of Hsp90 in different groups were shown in Figure 5. The expression level of Hsp90 increased significantly in radiated mice, but decreased to normal level after 5′-AMP administration before radiation. This is in close agreement with results of the proteomics analysis (Table 1) and highlights Hsp90 as a protein involved in the protection of 5′-AMP against irradiation in mice.

#### 2.4.2. Activity of Catalase

**Figure 6.**
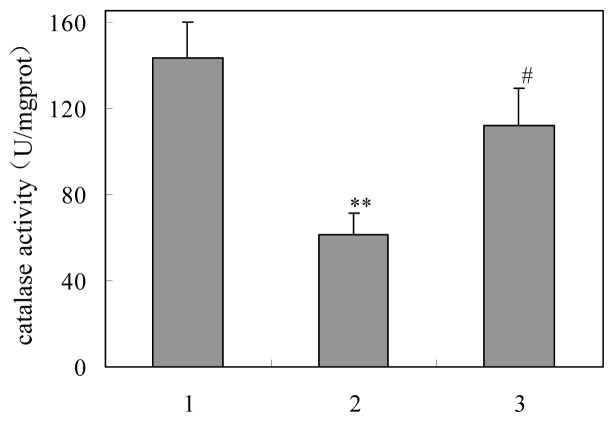
Effect of 5′-adenylic acid on catalase activity of liver in mice after γ-ray irradiation. (**1**) Normal group; (**2**) Model group; and (**3**) 5′-AMP-treated group. ** *p* < 0.01 *vs.* Normal; ^#^ *p* < 0.05 *vs.* Model.

The catalase activity of livers were obtained from three groups are shown in Figure 6. Significant decrease in catalase activity in single-irradiated mice was observed compared with normal mice (*p* < 0.01). The activity in group treated with γ-ray and 5′-AMP was elevated compared with the model groups (*p* < 0.05). The results was agreed with 2-DE.

### 2.5. Protection Mechanism Analysis of 5′-AMP against Radiation

By the proteomic technique, nine proteins, which may be molecular targets of 5′-AMP, were found in the present study. These proteins are involved in multiple cellular processes including cell differentiation and proliferation, oxidative stress and signal transduction and take place in several organelles including the cellular membrane, cytoplasm, nucleus, mitochondria and endoplasmic reticulum.

#### 2.5.1. Cell Viability and Proliferation

Two proteins involved in cell proliferation or apoptosis signals were both found to be up-regulated by 5′-AMP in radiated mice.

14-3-3 Protein epsilon was an important anti-apoptotic factor in cells that was involved in the regulation of cell apoptosis in many pathways. 14-3-3 Protein epsilon regulate the activity of key enzymes involved in transcription as MAPK kinase. For example, 14-3-3 protein epsilon shielded MEKK1 from caspase-3 that could split the kinase by the binding of 14-3-3 protein epsilon to the N-terminus regulated domain of MEKK1, thus inhibiting cell apoptosis. And, the 14-3-3 protein epsilon also participated in protein-protein interactions, for example, it interacted with Bad (a pro-apoptotic member of Bcl-2 proteins family) in a phosphoserine-dependent manner and inhibited the effect on promoting the cell apoptosis [4].

The other protein regulated by 5′-AMP was AI, which was the focal enzyme of the urea cycle hydrolyzing l-arginine to urea and l-ornithine. l-ornithine was a precursor of polyamines, which plays an important role in DNA synthesis, growth, gene replication and cell proliferation [5,6]. It is possible that the increased AI expression in 5′-AMP-treated mice, compared with single radiation-treated mice was associated with increased production of polyamines and higher rates of cell proliferation, thus protecting the mice against radiation induced damage.

#### 2.5.2. Ca^2+^ Binding

Annexins A5 belongs to a class of Ca^2+^-dependent membrane binding proteins. Annexin binds to both free Ca^2+^ and the phospholipids of the membrane structure [7] and might be a key moderator of cell apoptosis as a potent inhibitor of protein kinase C (PKC). PKC plays a significant role in the protein kinases of cell membranes. It affects protein Ser/Thr phosphorylation in cells and thus regulates cell differentiation and proliferation [8]. Annexin interacts with PKC in the presence of Ca^2+^ and phospholipids [9–11] and competition between annexin A5 and PKC for phospholipids [12] would be responsible for its inhibition activity.

Calmodulin (CaM) is calcium ion receptor and rich in non-muscle cell [13]. In eukaryotic cells, CaM can become active and play a role in cell growth and metabolism with many key enzymes (including adenylate cyclase, Ca^2+^-dependent phosphodiesterase and phosphorylase kinase) by binding with Ca^2+^ [14]. CaM increased the SOD activity by 50% in the presence of Ca^2+^ and scavenged free radicals, showing a protective effect against oxidative damage [15]. CaM also plays an important role in both G1/S and G2/M cell cycle progression [16]. Additionally, calcium (Ca^2+^) and calmodulin (CaM) are able to downregulate the Ras/Raf/MEK/ERK pathway allowing a proliferative signal [17].

In the present study, these two proteins were down-regulated in mice of the model group, which indicated radiation-induced cell injury and death. The expressions of the two proteins were recovered and approach that of the normal mice, when the mice received administration of 5′-AMP before irradiation. So, 5′-AMP perhaps protects the mice against radiation by cell apoptosis disruption and the restoration of cell proliferation.

#### 2.5.3. Oxidation-Reduction

Catalase is an endogenous antioxidant defense enzyme found in nearly all living organisms exposed to oxygen. It can eliminate hydrogen peroxide through catalyzing the decomposition of hydrogen peroxide to water and oxygen [18]. It is a very important enzyme in reproductive reactions. We detected downregulation of catalase in radiation-treated mice, however 5′-AMP upregulated the expression in AMP-treated mice. Due to the antioxidant nature of catalase, upregulation of this enzyme may reduce the free radicals generated from radiation and thus serves to protect cells or tissue from oxidative damage.

The other protein regulated by 5′-AMP was NAD-dependent Malate dehydrogenase (NAD-MDH), related to oxidizing and reduction reactions. The available evidence suggested that NAD-dependent malate dehydrogenase (MDH) was involved in the antioxidant role in preventing H_2_O_2_ or γ-radiation-induced damage through the action of oxaloacetate [19].

#### 2.5.4. Molecular Chaperone

Heat shock protein 90 (Hsp90) is an abundant protein that functions as a chaperone, thus preventing protein aggregation and helping denatured proteins to refold in an ATP-dependent fashion [20]. When cells reacted irritatively to internal or external cause, the expression of Hsps90 would consequently rise. Upregualtion of Hsp90 participated in tolerance against environmental stress and protected the injured cells, by the interaction with proteins with conformational changes responding to stimulus [21]. Additionally, Hsp90 is involved in cell survival in many biological processes: regulating surviving which plays double-acting role in cell multiplication and death; regulating Akt and Raf/MAPK which mediates cell survival and signal transduction; formulating a complex with Apaf-1 to disturb cell inherent caspase apoptosis pathway [22].

Protein disulfide isomerase (PDI) was the most versatile family member mainly located in the endoplasmic reticulum (ER), capable of catalyzing oxidation, reduction and disulfide isomerization [23]. It catalyzed correctly the disulfide formation during the protein folding in the ER. The ER showed responses stress when unfolded immature proteins accumulated. The ER withstood relatively mild stress through the regulation of expression of stress proteins such as PDI, although severe ER stress could result in apoptosis through ER-specific caspase-12. It was suggested that PDI assisted in the maturation and transport of unfolded secretory proteins as molecular chaperone and its upregulation had an essential role in cell survival under severe conditions [24].

We found compared with normal mice the expression of the two stress proteins in radiation-treated mice increases, but decreases in AMP-treated mice. It was considered that 5′-AMP may protect the mice against radiation by decreasing the stress by γ-radiation.

#### 2.5.5. Regulation of Muscle Contraction

Of nine proteins, one was related with inflammatory response, Tropomyosins (Tpm). Tpms are actin-binding proteins, and have more than 40 known isoforms which are generally classified into two groups based on apparent molecular mass: high *M*_r_ isoforms, e.g., Trm3; and low *M*_r_ isoforms, e.g., Trm5 [25]. Many researches have focused on the effect of Trm 3 on cancer cells migration [26–29].

Recently, there is solid evidence that the transforming growth factor beta (TGF-β) signaling pathway is a major cellular growth inhibitory and proapoptotic pathway in some cell types [30]. TGF-β could activate hepatic stellate cells to secrete abundant extracellular matrix constituents (ECM) that collectively form hepatic fibrosis in liver cirrhosis. The expression of tropomyosins mediated via Smad is required for TGF-β induction of stress fibers [31]. Tpm regulate actomyosin to control cytokinesis progression. Thus, Trm associated with hepatic fibrosis process.

In present study, we found upregulates expression of Trm3 in Model group compared to Normal. Considering the role of Trm on hepatic fibrosis as above, we hypothesized γ-rays probably caused hepatic fibrosis via the upregulated Trm3. 5′-AMP regulated the expression of this protein to about normal level, indicating it may relieve liver disease induced by radiation.

#### 2.5.6. Protection Pathway of 5′-AMP against Radiation

**Figure 7.**
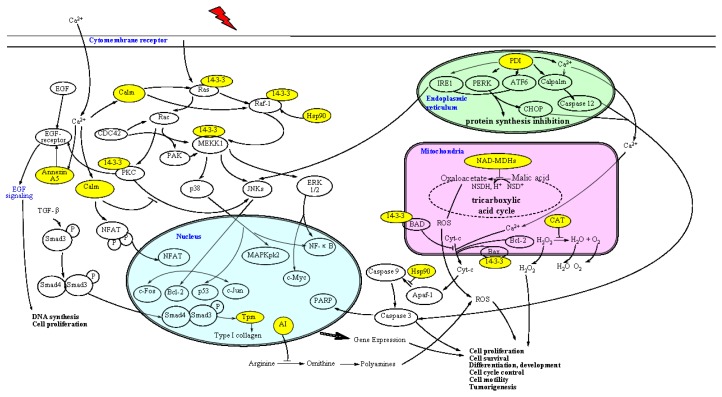
The inhibition pathway of 5′-AMP to cell apoptosis induced by γ-ray irradiation.

Based on these protein characteristic and functions, we analyzed the effect of exogenous nucleotides on its signal transduction pathway in γ-ray radiated mice and drew the map of its protection mechanisms (Figure 7); the primary ways are as follows:

(1) receptor pathway: To block Ras/Raf-1/MEKK1 signaling pathway through upregulation of 14-3-3 and calmodulin expression; to inhibit the PKC to activate and then catalyze the phosphorylation of threonine (Thr) residues on EGF receptor through upregulation of annexin A5. EGF was activated by combination with a receptor with tyrosine protein kinase activity, ultimately leading to DNA synthesis and cell proliferation [32]. Moreover, it is hypothesized that 5′-AMP inhibits cell apoptosis processes through upregulation of Hsp90 protein expression.
(2) mitochondrial pathway: *In vivo* by upregulating catalase and mitochondrial NAD-dependent malate dehydrogenase expression to improve the vitality of oxidoreductases, generate endogenous reducing substances, eliminate free radicals and maintain the structural integrity of cell membranes. The decrease of free radicals to reduce the release of cytochrome c (Cyt-c), preventing an enzyme called Caspase from launching an apoptotic signaling cascade, thereby reducing the radiation-induced oxidative damage and improving the body’s tolerance to radiation.
(3) endoplasmic reticulum pathway: In response to ER stress, cells have developed a self-protective signal transduction pathway termed the unfolded protein response (UPR). When external stimuli are light, three transcriptons (IRE1, PERK and ATF6) will be activated by PDI to deposit and degrade unfolded and misfolded proteins by UPR for the recovery of ER. Otherwise, specific signaling molecules (CHOP, JNK and Caspase 12) will be activated to induce cell apoptosis. In the present study, 5′-AMP upregulates the expression of the PDI protein, reflecting that the ER stress reaction decreased and apoptosis induced by ionizing irradiation was suppressed in the cells.

Thus the protective effects of nucleotides on radiation are various, influence each other and promote each other, finally to achieve the effect of anti-radiation.

## 3. Experimental Section

### 3.1. Experimental Agents

5′-AMP was sourced from Kaisheng Limited Co. (Nanjing, China) with purity ≥ 99% by HPLC. All reagents used in 2-DE were purchased from Bio-Rad Laboratories Inc. (Hercules, CA, USA), except the protease inhibitor (cOmplete ULTRA Tablets, Mini, EDTA-free, EASYpack, Roche, Basel, Switzerland) was from Roche (Basel, Switzerland). Other chemicals, except where specially noted, were purchased from Sigma-Aldrich Chemical Co. (St. Louis, MO, USA).

### 3.2. Experimental Animals

Male ICR mice (6–7 weeks old, 20–22 g) were obtained from Harbin Veterinary Research Institute, Chinese Academy of Agricultural Science (CAAS, license number: SCXK (HEI) 2006–2009, Harbin, China). The animal experimental protocol was approved by the local Institutional Animal Care and Use Committee (IACUC).

### 3.3. Irradiation

Whole-body ^60^Co γ-irradiation was performed at the Institute of Application of Atomic Energy, Heilongjiang Academy of Agricultural Sciences, Harbin, China. The tested animals were irradiated at an acute single dose of 4 Gy delivered at 95 cm source-to-skin distance. The dose rate was 1.0 Gy/min. The dose was determined based on the relative researches [33,34] and our preliminary experiment.

### 3.4. Experimental Design

After acclimation, thirty ICR mice were randomly divided into three experimental groups as follows, each consisting of ten mice:

Group I: Normal (no radiation + no treatment)
Group II: Model (radiation alone)
Group III: Radiation + 5′-AMP (radiation + treatment)

5′-AMP was administered using deionized water. The animals in groups I–II orally received deionized water only. Group III were administered with 5′-AMP at 0.16 g/kg·bw/d, which was determined according to the appendix of subdivision two of Chinese Pharmacopoeia (2010 vision) and national evaluation procedure for health foods [35]. After two weeks of intra-gastric administration, the animals were exposed to ^60^Co γ-ray (4 Gy) for whole-body radiation.

### 3.5. DNA Ladder Assay

DNA was isolated from liver tissue by using the methods of Khalaf *et al.* [36] to detect the apoptosis of the liver cell of three groups. One microgram DNA of mice from same groups was loaded in each well of 1.2% agarose gel containing 0.5 μg/mL ethidium bromide (EB) including 0.5 μg DNA marker (Takara Biotechnology Co., Ltd., Dalian, China). The electrophoresis was carried out in TAE buffer (40 mM Tris-acetate + 1 mM EDTA) using Mini-Sub Cell GT Cell agarose gel electrophoresis system (Bio-Rad, Hercules, CA, USA). After electrophoresis (4 V/cm) for 1.5 h, gel was studied under Gel Doc system (Bio-Rad, Hercules, CA, USA) and was photographed using a digital camera.

### 3.6. Protein Extraction and 2-DE

At 24 h post exposure, the mice were sacrificed. Liver samples were surgically removed immediately. The samples from animals of the same group were combined, and ground with liquid nitrogen before 2-DE analysis. Detergent soluble proteins were extracted from the combined sample by incubation in prepared lysis buffer including 7 M urea, 2 M thiourea, 65 mM DTT, 4% CHAPS, 0.2% Bio-lyte (pH 3–10) (*w*/*v*) and one protease inhibitor cocktail tablet which was added per 10 mL incubation solution. Homogenization of the sample was achieved by ultrasonication on ice for 15 min. The sediment was removed by centrifuging the homogenates at 12,000× *g* for 10 min at 4 °C. The protein content in supernate was determined by the Bradford method (Beyotime Institute of Biotechnology, Shanghai, China) using bovine serum albumin as standard. The protein samples were subpackaged and stored at −80 °C before use.

Protein samples of each group were desalinized strictly according to the instructions of the 2-D cleanup kit (ReadyPrep, Bio-Rad, Hercules, CA, USA). 2-DE was carried out using Bio-Rad 2-DE system (PROTEAN IEF cell, Bio-Rad) following the Bio-Rad Laboratories handbook. Briefly, each protein sample (800 μg) was applied for IEF using the ReadyStrip IPG Strips (17 cm, pH 3–10, Bio-Rad). The strips were placed into a Protein IEF cell and were rehydrated at 50 V for 12–16 h (20 °C). After that, the proteins were separated based on their isoelectric points according to the following protocol: 250 V with linear climb for 30 min; 1000 V with rapid climb for 60 min; 10,000 V with linear climb for 5 h and 10,000 V with rapid climb until 80,000 Vh was reached. After IEF, the IPG strips were equilibrated for 10 min in a buffer containing 0.375 M Tris-HCl (pH 8.8), 20% glycerol, 6 M urea, 2% SDS and 2% DTT, followed by further treatment in a similar buffer (but containing 2.5% iodoacetamide instead of DTT) for 10 min, and then directly applied onto 12% homogeneous SDS-PAGE gels for electrophoresis using a Powerpac Basic electrometer (Bio-Rad). For protein samples from each group, triplicate electrophoresis was performed to ensure reproducibility.

### 3.7. Staining and Image Analysis

The gels were stained using coomassie brilliant blue G-250 (Bio-Rad) overnight and then properly destained for about 2 h. Digitized images of two-dimensional gels were generated using Laboratories GS-700 Densitometer (Bio-Rad and the dots were quantitated by PDQuest 2-D analysis software (Ver. 7.41, Bio-Rad) to obtain the total valid spots and differential expression of proteins number. Each of the significantly differentially expressed protein spots was excised from the gels and placed into an eppendorf tube respectively. Gel pieces were delivered to Shanghai GeneCore BioTechnologies Co. Ltd., (Shanghai, China) for identification.

### 3.8. In-Gel Tryptic Digestion

Gel pieces were washed three times in ddH_2_O, then destained with a solution (50% acetonitrile (ACN)/25 mmol/L ammonium bicarbonate, pH 8.0) to colorless transparent and dried by vacuum drying method until completely dry. The samples were then swollen in 20–25 μL digestion buffer (10–15 μg/mL Trypsin and 25 mmol/L ammonium bicarbonate, pH 8.0). After 15 min of incubation, the gels were digested more than 15 h at 37 °C. 0.75 μL hydrolyzed protein sample was mixed with the same amount of MALDI matrix (10 mg/mL α-cyano-4-hydroxycinnamic acid) and spotted onto the MALDI target plates.

### 3.9. MALDI-MS/MS Analysis and Protein Identification

Mass analysis was performed on a 4700 Proteomics Analyzer with delayed ion extraction (Applied Biosystems, Foster City, CA, USA). Mass spectra were obtained in a mass range of 700–3500 Da, using a laser (355 nm, Nd:YAG) as desorption ionization source. The precursor ions were selected automatically for the MS/MS analysis. The accelerated voltage was operated at 20 kV and the positive ion mass spectra were recorded. MS accuracy was externally calibrated with trypsin-digested peptides of myoglobin. All data analysis and database searching were performed by the GSP Explorer software with the search engine MASCOT (Matrix Science, London, UK) against NCBInr protein sequence database. Search category was mouse. The peptide mass tolerance was set at 100 ppm, and MS/MS tolerance was 0.8 Da. The maximum number of missed cleavages was set to 1 with trypsin as the protease. During the database retrieval, the peaks of pancreatin self-degradation and pollutants were eliminated artificially.

### 3.10. Western Blot Validation

Protein samples (50 μg) were electrophoretically separated on a SDS-PAGE gel at 250 V using Powerpac Basic electrometer (Bio-Rad) following the Bio-Rad Laboratories handbook. Protein from the gel was transferred into a nitrocellulose (NC) membrane paper at 100 V for 2 h using a Fast-Transfer Blot System (Bio-Rad). Strips were washed three times in TBST solution. Dried skim milk blocking solution (5%) was added to the membrane and incubated on a rocker for 1 h. A 1:200 dilution of rabbit polyclonal antiHSP-90 primary antibody (Boster Biological Technology., LTD., Wuhan, China) and 1:500 dilution of rabbit polyclonal antiactin primary antibody (Boster Biological Technology., LTD., Wuhan, China) was added and incubated at 4 °C overnight. The strip was rinsed three times in TBST solution and incubated with a 1:500 dilution of antirabbit IgG alkaline phosphatase (AP) secondary antibody (Sigma-Aldrich Co., St. Louis, MO, USA) for 1 h on a rocker. The strip was rinsed once more and colorometrically developed using Western blue stabilized substrate for AP. The gel was scanned using a Gel Doc scanner (Bio-Rad) and densitometry analyses carried out with Quantity One 1-D analysis Software (Bio-Rad). Expression of target proteins in each sample from three groups was internally normalized to β-actin. The test was performed in triplicate.

### 3.11. Activity of Catalase

Liver sample was ground with normal saline (NS) to 10% homogenate on ice after being exactly weighed. The supernatant was collected by centrifuging at 4000 rpm for 10 min at 4 °C. Activities of catalase in liver tissue were measured using commercial kits (Nanjing Jiancheng Bioengineering Institute, Nanjing, China).

### 3.12. Statistical Analysis

All the experiments were performed in triplicate. The results were expressed as mean ± standard deviation and statistical significance was determined by One-way ANOVA (LSD) employing the computer SPSS statistic package (Ver. 17.0, SPSS Inc., Chicago, IL, USA). *p* < 0.05 was considered significant.

## 4. Conclusions

To date, this study was the first to employ the proteomic technique to search globally for the proteins of liver from radiated mice influenced by 5′-AMP. Nine proteins, which might be molecular targets of 5′-AMP, were identified with an MALDI-TOF MS/MS analyzer in the present study. The changes in the expression of several proteins that we have documented after 5′-AMP reflect the involvement of various regulatory pathways. To make clear this mechanism, future investigations will focus on establishing conclusively the molecular roles of identified proteins in protective response with 5′-AMP using knock-out mice, neutralizing antibodies, and/or siRNA approaches, and continuing to identify other additional differentially expressed proteins and relative proteins in the important pathway, such as MAPK, PKC, Caspase 9 and so on using MALDI-TOF MS/MS.

## Figures and Tables

**Table 1.** Expression and identification of the selected proteins in three groups.

Spot No.	Protein name	Protein expression		

		Normal group	Model group	AMP-treated group
201	Type I arginase (AI)	6860.4 ± 616.1	2543.8 ± 284.2 **	4127.3 ± 392.5 *,##
1207	Annexin A5	1086.4 ± 179.3	533.5 ± 39.5 **	1279.4 ± 189.6 ##
1220	Calmodulin (CaM)	2068.3 ± 218.5	67.3 ± 5.3 **	949.9 ± 34.9 **,##
7621	Catalase	1914.4 ± 183.0	1112.3 ± 109.3 **	1659.3 ± 259.2 #
227	Tpm3 protein	2354.5 ± 204.4	3204.6 ± 287.7 **	2059.5 ± 187.5 ##
3802	Pdia4 protein	112.3 ± 14.0	3365 ± 311.7 **	108 ± 9.1 ##
220	Tyrosine 3-monooxygenase/tryptophan 5-monooxygenase activation protein, epsilon polypeptide (14-3-3 Protein epsilon)	2405 ± 192.3	988.3 ± 105.9 **	1950 ± 195.2 *,##
202	NAD-Malate dehydrogenase (NAD-MDH)	2772.8 ± 211.9	1490 ± 101.9 **	3053.9 ± 271.3 ##
3801	Heat shock protein 90 (Hsp90)	1521.2 ± 148.6	2099.5 ± 228.4 **	1640.5 ± 96.9 #

** *p* < 0.01,

* *p* < 0.05 *vs.* Normal;

# *p* < 0.05,

## *p* < 0.01 *vs*. Model.
